# The impact of genetic variants of the IGF-1 axis on surgical outcomes and prognosis in ovarian cancer

**DOI:** 10.1007/s11033-026-11508-4

**Published:** 2026-02-04

**Authors:** Inês de Almeida Lopes, Mariana Moreira Pires, Deolinda Pereira, Valéria Tavares, Inês Guerra de Melo, Rui Medeiros

**Affiliations:** 1 Molecular Oncology and Viral Pathology Group, Research Centre of IPO Porto (CI-IPOP)/Pathology and Laboratory Medicine Dep., Clinical Pathology SV/RISE@CI-IPOP (Health Research Network), Portuguese Oncology Institute of Porto (IPO Porto)/Porto Comprehensive Cancer Centre (Porto. CCC), Porto, 4200-072 Portugal; 2 Clinical Pathology Service, Portuguese Institute of Oncology of Porto (IPO Porto), Porto, 4200-072 Portugal; 3 Faculty of Medicine,, University of Porto (FMUP), Porto, 4200-072 Portugal; 4 Department of Medical Oncology, Portuguese Institute of Oncology of Porto (IPO Porto), Porto, 4200-072 Portugal; 5 Research Department, Portuguese League Against Cancer (NRNorte), Porto, 4200-172 Portugal; 6 Faculty of Health Sciences, Fernando Pessoa University, Porto, 4200-150 Portugal

**Keywords:** Ovarian cancer (OC), Insulin growth factor 1 (IGF-1), Genetic polymorphism, Single-nucleotide polymorphisms (SNP), Biomarker

## Abstract

**Background:**

Ovarian cancer (OC) remains the most lethal gynaecological malignancy, largely due to late-stage diagnosis, tumour heterogeneity, and high recurrence rates. The insulin-like growth factor-1 (IGF-1) axis has been implicated in tumour proliferation, survival, and treatment resistance. Yet, the prognostic relevance of its genetic variants in OC is not well established. The present study aims to evaluate the impact of two IGF-1-related single-nucleotide polymorphisms (SNPs), *IGF1* rs6220 and *IGF1R* rs2016347, on the clinical outcome of 330 OC patients.

**Methods and results:**

SNP genotyping was performed using the TaqMan^®^ Allelic Discrimination methodology. Regarding *IGF1* rs6220, G allele carriers presented significantly improved overall survival compared with AA homozygotes within the subgroup of women undergoing suboptimal cytoreductive surgery (residual disease ≥ 1 cm) (*p* = 0.039). As for *IGF1R* rs2016347, the TT genotype was associated with shorter disease-free survival than A allele carriers within the well-differentiated tumour group (*p* = 0.028).

**Conclusions:**

These results indicate a context-dependent impact of IGF-1 axis polymorphisms on OC prognosis, suggesting their potential utility as molecular markers. Further validation in larger, independent cohorts, together with functional studies, is warranted to confirm these results and clarify the biological mechanisms underlying the influence of IGF-1-related genetic variants on OC behaviour.

## Introduction

Ovarian cancer (OC) is the most lethal gynaecological malignancy worldwide. In 2022, it was ranked as the third most prevalent gynaecological cancer and the eighth leading cause of malignancy-related mortality in women, accounting for approximately 5% of female cancer deaths [1]. Although OC represents only about 1% of new cancer diagnoses in women, it contributes significantly to global gynaecological cancer mortality, reflecting its disproportionate global burden [1, 2]. The poor prognosis of OC is primarily due to late-stage diagnosis, tumour heterogeneity, and acquired chemoresistance, resulting in five-year survival rates below 50% in most countries [3]. Current prognostic assessment relies on the International Federation of Gynaecology and Obstetrics (FIGO) stage, histopathological subtype and grade, serum cancer antigen 125 (CA-125), surgical cytoreduction status, and chemotherapy regimens [4–7]. However, these clinical indicators remain insufficient to fully arrest disease complexity, highlighting the need for novel molecular biomarkers to refine prognosis, guide personalised therapy, and improve disease monitoring [8, 9].

Surgery is a key determinant of survival in the management of OC, as it allows definitive diagnosis, accurate staging, prognostic assessment, and optimal cytoreduction of tumour, thus enhancing the efficacy of subsequent chemotherapy [10]. The primary surgical goal is “optimal” cytoreduction, typically defined as leaving residual disease measuring 1 cm or less, as maximal tumour removal has been strongly associated with improved chemotherapy response and survival outcomes. Indeed, residual disease after cytoreductive surgery has emerged as the most important prognostic factor [11–14]. These findings underscore that, although medical treatment alone rarely achieves a cure, optimised surgical management is essential for improving survival in OC patients [15].

Cellular differentiation also plays an important role in OC biology. It is the process by which healthy cells develop specific structures and functions to perform their role in the body [16, 17]. In the case of OC, well-differentiated tumour cells resemble healthy ovarian tissue, growing more slowly and being less aggressive than poorly differentiated tumours. Grading provides important prognostic information, including tumour behaviour, therapy response, and patient survival. Clinically, well-differentiated OC is associated with longer overall survival (OS) but is relatively chemo-resistant and more challenging to treat upon disease recurrence [18, 19].

Within the context of tumour metabolism, insulin-like growth factor 1 (IGF-1) is one of the most studied signalling molecules due to its crucial role in the physiology of tumour progression and therapy resistance. IGF-1 is produced in the liver by stimulation from the pituitary growth hormone (GH). The binding of IGF-1 to its receptor (IGF1R) triggers autophosphorylation of tyrosine residues, initiating downstream signalling. Dysregulation of IGF-1/IGF1R signalling activates PI3K-Akt-mTOR and mitogen-activated protein kinase (MAPK) cascades, promoting cell proliferation, survival, and angiogenesis [20–22]. Considering the involvement of the IGF-1 pathway in cancer biology, the study of its related genetic polymorphisms, particularly single-nucleotide polymorphisms (SNPs), is notably relevant. SNPs, defined as variations at a single nucleotide base, represent the most frequent form of DNA variation and can significantly influence gene expression and protein function, thereby modulating disease susceptibility [23]. Specifically, these genetic variants may modulate the activity of proteins in the IGF-1 axis, affecting clinical outcomes in OC patients, although the contribution of these variants remains poorly explored. In recent years, several SNPs related to the IGF-1 signalling pathway have been identified, among which *IGF1* rs6220 and *IGF1R* rs2016347 are two of the most studied [24–26].

Given the high incidence of OC and its significant impact on patient survival, investigating the genetic variants linked to the *IGF-1* axis is both biologically and clinically important [27]. Thus, this study aimed to assess the influence of *IGF-1* rs6220 and *IGF1R* rs2016347 and their association with clinical predictive and prognostic factors, for a better understanding of disease progression and treatment response in OC.

## Methods

### Population description and data collection

A retrospective cohort study included Caucasian OC patients who were admitted at the Gynaecology Clinic of the Portuguese Institute of Oncology of Porto (IPO Porto) between January 1996 and December 2012, for their first-line cancer treatment. The standard treatment protocol involved cytoreductive surgery and platinum-based chemotherapy. Exclusion criteria comprised being under 18 years of age, those admitted only for a second opinion, individuals who underwent specific treatment techniques, patients whose follow-up was conducted at another institution, and those who refused to provide informed consent. The selected cohort encompassed 330 patients with confirmed OC, from whom peripheral blood samples were available in the institutional biobank.

All OC cases were staged in accordance with the FIGO system, and the tumour response to chemotherapy was assessed using the Rustin criteria [28, 29]. Information on patients’ follow-up, including demographic and clinical data, was retrieved from their electronic medical files.The mean follow-up time in the study was 144.4 months (median = 148.0 months).

Briefly, the mean age of the patients, which also corresponds to the median age, was 55 years. Most of the patients (65%) were post-menopausal and were diagnosed at advanced cancer stage (FIGO III/IV; 60%). Regarding the histological subtype, 57% of the patients were diagnosed with serous tumours, 13% with clear cell, 10% with endometrioid, 10% with mucinous, and the remaining with less common subtypes. Well-differentiated tumours represent 20% of the cases, constituting a minor proportion within the cohort. Concerning therapeutic management, most patients received standard treatment (93%), consisting of cytoreductive surgery followed by chemotherapy with paclitaxel combined with carboplatin or cisplatin. Complete surgical resection was achieved for 53% of the patients. Cytoreduction to < 1 cm residual disease was obtained in 3% of the patients, whereas residual disease ≥ 1 cm was observed in 32% of patients. Exploratory surgery without cytoreductive intent was performed in 12% of the patients.

This study has been approved by the ethics committee of IPO Porto (CES IPO:286/2014). All patients provided written informed consent according to the principles of the Helsinki Declaration.

### Sample processing and genomic DNA isolation

Using a standard phlebotomy technique, peripheral venous blood samples were collected from patients and placed into anticoagulant ethylenediaminetetraacetic acid (EDTA)-coated tubes before initiation of first-line chemotherapy. Genomic deoxyribonucleic acid (DNA) was isolated from the blood samples of each patient for SNP genotyping using the QIAamp DNA Blood Mini Kit (Qiagen^®^ 51106, Hilden, Germany), following the manufacturer’s protocol. DNA purity and concentration were assessed using the NanoDrop Lite spectrophotometer (Thermo Fisher Scientific^®^, Waltham, MA, USA). After extraction and quantification, all DNA samples were stored at -20 °C until further analysis.

### SNP selection and genotyping

A literature review was conducted to identify relevant SNPs associated with tumour progression and cancer prognosis. Priority was given to genetic variants with reported roles and/or relevance to cancer patients’ prognosis. After a comprehensive literature review, polymorphisms were selected based on the following criteria: (i) located in genes related to the IGF-1 pathway; (ii) with known functional impact on encoded proteins’ activity, (iii) with previously roles reported in cancer; (iv) minor allele frequency (MAF) ≥ 10% to ensure adequate representation of all genotypes in study; and (v) availability of TaqMan^®^ genotyping assays. Linkage disequilibrium (LD) among the selected genetic variants was also evaluated to avoid overlapping effects. Applying these criteria, *IGF1* rs6220 and *IGF1R* rs2016347 emerged as the SNPs with the most cumulative evidence.

SNP genotyping was performed using a StepOnePlus™ Real-time Polymerase Chain Reaction (RT-PCR) system (Applied Biosystems^®^, Carlsbad, CA, USA), applying a TaqMan allelic discrimination method. Each PCR reaction was conducted in a total volume of 6.0 µL, consisting of 2.5 µL of TaqPath™ ProAmp™ Master Mix (1×), 2.375µL of nuclease-free water, 0.125 µL of the predesigned TaqMan^®^ Genotyping Assay for *IGF1* rs6220 (C___2801119_10) and *IGF1R* rs2016347 (C___8723111_20), as well as 1.0 µL of genomic DNA. Negative controls (without genetic material) were included in each PCR run to monitor false positives. Thermal cycling conditions for DNA amplification were as follows: activation of Taq DNA polymerase (10 min at 95 °C), followed by denaturation (15s of 45 cycles at 95 °C), and pairing and extension of primers (60s at 60 °C). To ensure genotyping quality and reproducibility, at least 10% of the samples were randomly selected for duplicate analysis.

Allelic discrimination and amplification data were analysed using StepOne Software v2.3 (Applied Biosystems^®^, Foster City, CA, USA). Genotyping results were independently reviewed by three researchers, each blinded to patients’ clinical and pathological data.

### Statistical analysis

Data analysis was performed using Statistical Package for the Social Sciences (SPSS) for Windows (version 30.0, IBM Corp., Armonk, NY, USA).

Data distribution was tested through the Kolmogorov-Smirnov test (*N* > 50). Continuous variables were described using the mean value as the cut-off for normally distributed data or the median for non-normally distributed data.

Genotype distribution of each SNP was compared with that reported for the Iberian population in the Ensembl database (https://www.ensembl.org/index.html, accessed on 22 September 2025). Hardy-Weinberg equilibrium (HWE) was evaluated using the chi-square test (χ^2^) to assess deviations from expected genotype frequencies. Associations between the SNPs and patients’ clinical and demographic characteristics (categorical variables) were also evaluated employing the χ^2^ test.

The impact of the SNPs on the patient’s clinical outcomes, including progression-free survival (PFS), disease-free survival (DFS), and OS, was evaluated. PFS was defined as the time from the start of cancer treatment to the date of progression, related mortality, or the patient’s last clinical evaluation. DFS, on the other hand, referred to the time interval between the date of diagnosis and either the date of first recurrence or of the last clinical evaluation for patients who achieved a complete response to first-line treatment. Regarding OS, it was defined as the period from the patient’s diagnosis until death, from any cause, or the last clinical assessment. Tumour progression was assessed using the Response Evaluation Criteria in Solid Tumours (RECIST) version 1.1 [30]. Survival curves were generated using the Kaplan–Meier method, and survival probabilities were calculated using either the log-rank test or the Tarone-Ware test, based on assessment of the proportional hazards assumption. The most appropriate genetic model for each SNP (dominant or recessive) was established by evaluating the Kaplan-Meier curves under the additive genetic model. Subgroup analyses were conducted according to histological subtype (serous vs. non-serous; serous vs. clear cell vs. endometrioid vs. mucinous vs. others), patient age at OC diagnosis (< 55 vs. ≥55 years), FIGO stage (I/II vs. III/IV), tumour grade (well-differentiated vs. others), and extent of surgical resection (complete vs. others). Only the significant findings were presented. Additionally, the impact of *IGF1* rs6220 and *IGF1R* rs2016347 on the risk of tumour recurrence, progression, and patient death was estimated through a multivariable Cox proportional hazards model, adjusted for the same patient demographic and clinicopathological factors. Once again, only the significant results were described. All the tests were two-sided, considering a statistically significant result at *p* < 0.05.

## Results

### Distribution of SNP genotypes

The distribution of the SNP genotypes is represented in Table 1. When compared to the estimated frequencies in the Iberian population, both *IGF1* rs6220 and *IGF1R* rs2016347 were in HWE (χ^2^ test, *p* = 0.726 and *p* = 0.735, respectively), demonstrating no significant deviation from expected genotype frequencies.

**Table 1:** Genotype distribution of the SNPs compared with the reference Iberian population

SNP	Genotype	MAFi 1* (MA)	*N* i * (%)	MAFs (MA)	*N* s (%)
***IGF1*** **rs6220**	AA	29.9% (G)	54 (50.5)	28.5% (G)	163 (50.5)
	AG		42 (39.3)		136 (42.1)
	GG		11(10.3)		24 (7.4)
***IGF1R*** **rs2016347**	TT	42.5% (G)	35 (32.7)	45.9% (G)	100 (30.4)
	TG		53 (49.5)		156 (47.4)
	GG		19 (17.8)		73 (22.2)

*According to the Ensembl database accessed on 22nd September 2025. Abbreviations: MA, minor allele; MAFs, minor allele frequency in the study cohort; MAFi, minor allele frequency in the Iberian population; Ni, total number in the Iberian population; Ns, total number in the study cohort; SNP, single-nucleotide polymorphism

### Associations between the SNPs and patients’ characteristics

In the overall cohort, no significant associations were found between the genetic variants and patients’ demographic and clinicopathological features, including histological subtype (serous vs. non-serous; serous vs. clear cell vs. endometrioid vs. mucinous vs. others), patient age at tumour diagnosis (< 55 vs. ≥55 years), FIGO stage (I/II vs. III/IV), tumour grade (well-differentiated vs. others), extent of surgical resection (complete vs. others) and hormonal status (pre- vs. post-menopausal) (χ^2^ test, *p* > 0.05).

### Associations between the SNPs and patients’ prognosis

Considering the entire cohort, no significant impact of the *IGF1* rs6220 and *IGF1R* rs2016347 genotypes on patients’ PFS, DFS, and OS was observed, independently of the genetic model (log-rank test and the Tarone-Ware test, *p* > 0.05). Likewise, stratifying the analysis according to histological subtype (serous vs. non-serous; serous vs clear cell vs. endometrioid vs. mucinous vs. others), patient age at OC diagnosis (< 55 vs. ≥55 years), and FIGO stage (I/II vs. III/IV), did not reveal significant associations (log-rank test and the Tarone-Ware test, *p* > 0.05).

**Fig. 1:**
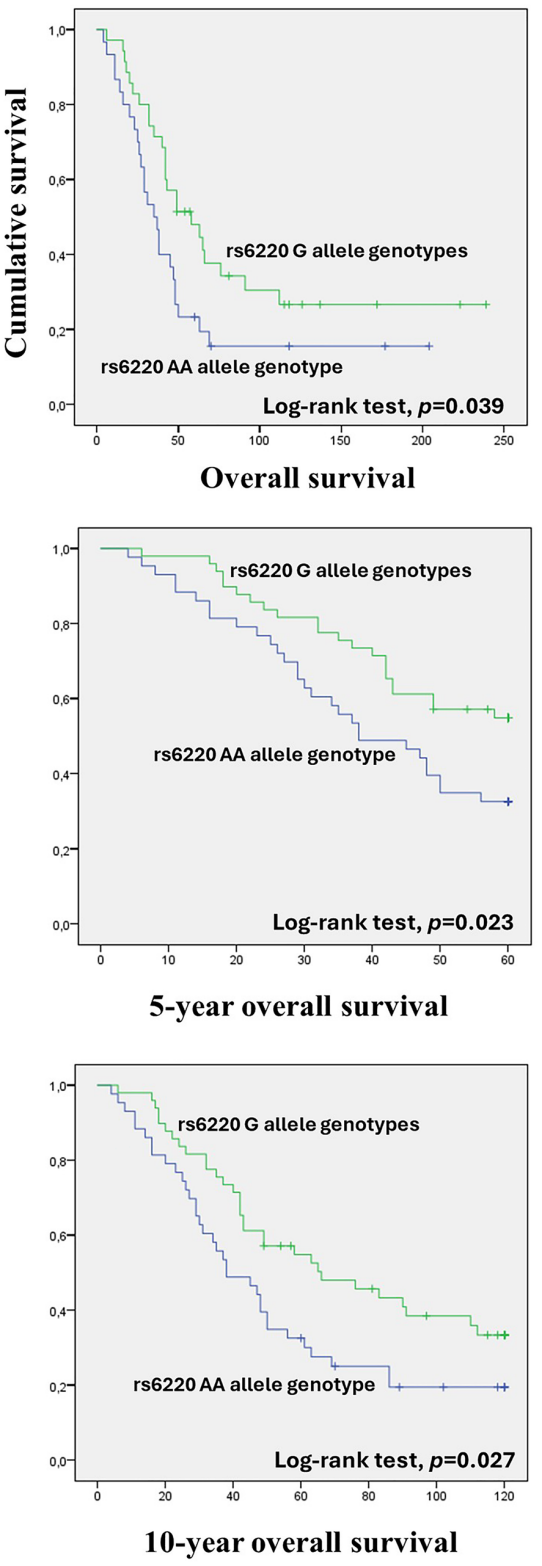
Association between *IGF1* rs6220 polymorphism and patients’ survival. Overall survival (OS) (**a**), 5-year OS (**b**), and 10-year OS (**c**) by Kaplan–Meier and log-rank test among OC patients who underwent suboptimal surgery (residual disease ≥ 1 cm). Patients with G allele genotypes (GG and AG) had higher OS, 5-year OS, and 10-year OS compared to patients with the AA genotype (*p* = 0.039, *p* = 0.023, and *p* = 0.027, respectively)

When stratifying the analysis according to the extent of surgical resection, a significant impact of *IGF1* rs6220 on OS was observed among patients with >1 cm residual disease after surgery. Those carrying the G allele genotype exhibited a better OS compared to the AA genotype (AG/GG vs. AA; log-rank test, *p* = 0.039; Fig. 1a). Namely, G allele carrier patients presented a mean OS of 97.88 ± 15.66 months, whereas the observed mean OS for AA genotype patients was 58.80 ± 11.96 months. The protective effect of the G allele was even more evident when considering 5 and 10-year OS (log-rank test, *p* = 0.023 and *p* = 0.027; Fig. 1b and **c**, respectively). Consistently, in the corresponding Cox analyses considering the same patient subgroup, those with the AA genotype showed a significantly increased risk of death compared to G allele carriers. Specifically, for total OS and 10-year OS, the AA genotype showed a similarly increased risk compared to G allele carriers [hazard ratio (HR) = 1.79, 95% CI = 1.02–3.16, *p* = 0.043; and HR = 1.78, 95% CI = 1.02–3.16, *p* = 0.043, respectively]. This effect was even more pronounced when considering 5-year OS, where the AA genotype exhibited an approximately twofold increased risk of death compared to G allele carriers [HR = 2.03, 95% CI = 1.09–3.78, *p* = 0.025]. No significant association was detected in the multivariable Cox analysis. Likewise, no significant impact of the *IGF1* rs6220 on patient DFS and PFS was observed in all the subgroup analyses (log-rank test and the Tarone-Ware test, *p* > 0.05).

**Fig. 2:**
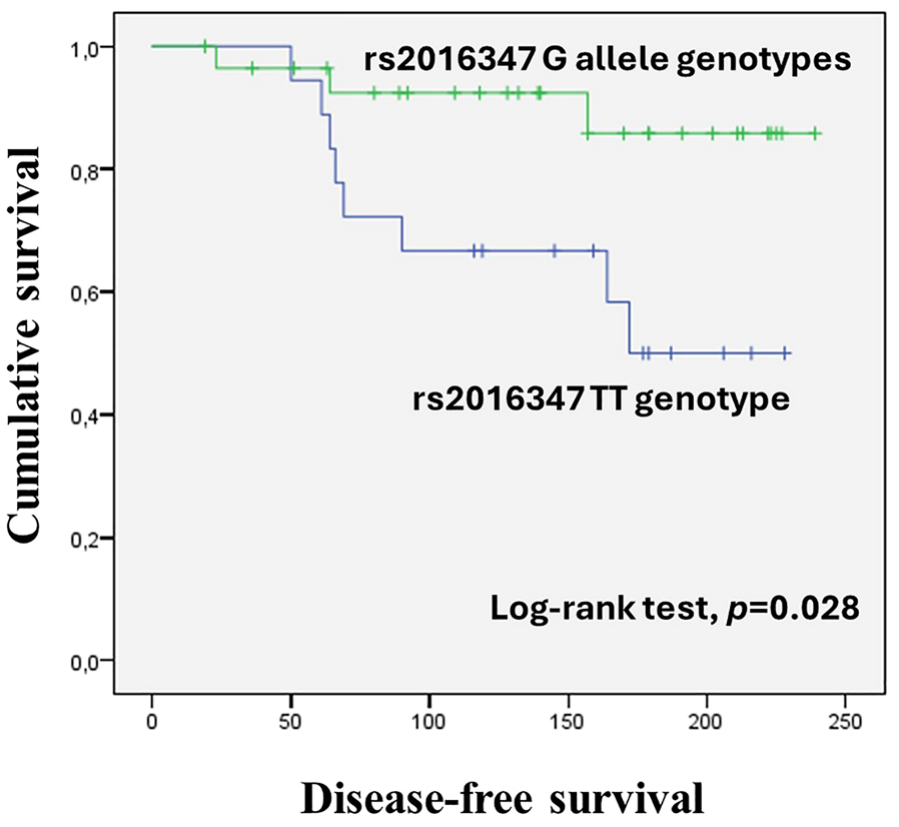
Association between *IGF1R* rs2016347 polymorphism and patients’ survival. Disease-free survival (DFS) by Kaplan-Meier and Tarone-Ware test for OC patients with well-differentiated tumours. Patients with the TT genotype had lower DFS compared to patients with G allele genotypes (*p* = 0.028)

Regarding the *IGF1R* rs2016347 polymorphism, a significant impact of the TT genotype on DFS was observed for patients with well-differentiated tumours (Tarone-Ware test, *p* = 0.028; Fig. 2). Namely, TT genotype patients exhibited a short time until disease recurrence when compared to G allele genotype patients (mean DFS of 164.22 ± 17.46 and 218.84 ± 11.05 months, respectively). Moreover, in a univariate Cox analysis for the risk of recurrence of OC patients, patients with the TT genotype had a fourfold increase in the risk of recurrence compared to patients with G allele genotypes [HR = 4.09, 95% CI = 1.09–15.43, *p* = 0.037]. Important predictors for the risk of OC recurrence, namely patients’ age at OC diagnosis (< 55 vs. ≥55 years), FIGO staging (I/II vs. III/IV), and tumour histology (serous vs. non-serous), were further explored in a multivariate Cox analysis in the subgroup of well-differentiated tumours to assess the independent predictive value of the *IGF1R* rs2016347 polymorphism. Using the backward Wald method, the genetic variant emerged as the most significant predictor of disease progression across all three groups (Table 2). No significant impact of the *IGF1R* rs2016347 on patient PFS and OS was observed in the subgroup analyses (log-rank test and the Tarone-Ware test, *p* > 0.05).

**Table 2:** Multivariable Cox analysis on the risk of tumour recurrence of OC patients with well-differentiated tumours (*N* = 53)

Variable	aHR	95% CI	*p*-value
*IGF1R* rs2016347 (TT vs. GG/GT^1^)	3.89	**1.01–14.74**	**0.05**
Age at OC diagnosis (< 55 vs. ≥55 years^1^)	0.28	0.12–3.40	0.60
FIGO staging (I/II vs. III/IV^1^)	0.86	0.49–7.25	0.35
Tumour histology (non-serous vs. serous^1^)	0.47	0.17–2.39	0.49

Bold results were deemed significant. 1 - Reference group. Abbreviations: aHR, adjusted hazard ratio; CI, confidence interval; N, number of participants; OC, ovarian cancer

## Discussion

Despite significant advances in cancer management, long-term survival in OC remains limited, largely due to late-stage diagnosis and the frequent development of resistance to treatment. These challenges not only worsen patient prognosis but also increase the risk of complications [3, 5, 31–33]. Emerging evidence suggests that identifying optimised prognostic biomarkers is fundamental for improving clinical management and survival in patients with OC [34]. Nevertheless, the prognosis may be influenced by biological pathways that modulate tumour behaviour and treatment response. Among these, the IGF-1 axis has attracted particular attention as IGF-1 and its receptor, IGF1R, appear to be relevant mediators in the context of ovarian carcinogenesis [22, 27, 35]. Building on this rationale, the present study investigated the influence of two IGF-1 axis SNPs, *IGF1* rs6220 and *IGF1R* rs2016347, on the clinical outcome in an Iberian OC population, to identify clinically important prognostic markers of the disease.

Focusing on the evaluated SNPs, *IGF1* rs6220 (A > G) is a genetic variant located in the 3′ untranslated region (3′UTR) of the *IGF1* gene, modulating its expression. Specifically, the G allele has been described to potentially increase IGF-1 expression levels. In turn, elevated IGF1 levels are known to contribute to cancer progression, resistance to therapy, and the establishment of a pro-metastatic microenvironment [24–26, 36]. In contrast, in the present study, the G allele was found to be significantly associated with better OS, among patients who underwent suboptimal surgery. Those with the *IGF1* rs6220 G allele genotypes survived longer periods compared with their counterparts. Although supported by limited existing evidence, these findings suggest that this allele may stimulate the activity of the IGF-1 mediated signalling cascades that support endothelial and metabolic homeostasis and reduce inflammation. Namely, through activation of the PI3K-Akt-mTOR pathway, IGF1 promotes protein synthesis, tissue regeneration, muscle mass preservation, and metabolic balance [34–40]. Importantly, this signalling also enhances nitric oxide (NO) production, supporting the regulation of inflammatory processes and contributing to the maintenance of endothelial integrity [41]. Simultaneously, stimulation of the Raf-MAPK pathway promotes angiogenesis, cell repair, and endothelial function, further reinforcing the potential protective role of IGF-1 signalling in this context [22, 42]. Collectively, these effects may translate into beneficial effects on key postoperative complications, thereby promoting improved functional recovery and conferring a survival advantage in OC patients with significant residual disease, ultimately underscoring the paradoxical nature of IGF-1.

Regarding the *IGF1R* rs2016347 (T > G), this variant is located in the 3’UTR of the *IGF1R* gene, affecting IGF1R expression and circulating levels. According to the literature, the T allele is associated with downregulation of IGF1R expression compared with the G allele, leading to reduced activation of the IGF1R signalling pathway [24, 43, 44]. In the present study, among patients with well-differentiated OC, those carrying the *IGF1R* rs2016347 TT genotype showed a higher risk of recurrence when compared with carriers of the G allele. This finding may be partially explained by the distinct biological behaviour of well-differentiated tumours, which are generally less aggressive and display lower proliferative activity, but also tend to exhibit reduced sensitivity to chemotherapy, potentially influencing OC recurrence dynamics [19, 45]. Functionally, IGF1R downregulation associated with the TT genotype may attenuate PI3K-Akt-mTOR signalling, indirectly leading to decreased NO synthesis, and consequently metabolic instability, and a pro-inflammatory microenvironment that favours tumour recurrence. Thus, in well-differentiated OC, where the tumour cells rely on tightly regulated growth and survival pathways, the reduced IGF1R expression linked to the TT genotype may paradoxically worsen prognosis by compromising homeostatic mechanisms and promoting inflammation-driven recurrence [22, 46].

Future research should aim to externally replicate and validate these results in larger cohorts and diverse populations to assess the generalisability of the findings and provide deeper insights. Also, prospective studies should be conducted in order to assess other clinical factors that may constitute confounding factors. Moreover, functional studies are also required to better understand the molecular mechanisms underlying these results.

## Conclusion

Over recent years, the identification of reliable prognostic biomarkers has become a major focus to improve OC management and patient outcomes. Within cancer research, markers related to the IGF-1 axis have attracted considerable attention due to their involvement in tumour progression and angiogenesis [34, 46, 47]. In this study, we evaluated the influence of *IGF-1*-related genetic variations on OC prognosis. The *IGF1* rs6220 G allele genotype and *IGF1R* rs2016347 TT genotype were significantly associated with OS and DFS, respectively. Namely, the *IGF1* rs6220 G allele genotypes correlated with better OS among patients who underwent suboptimal surgery, whereas the *IGF1R* rs2016347 TT genotype was linked to an increased risk of recurrence among those with well-differentiated tumours, supporting the hypothesis of an indirect protective role of IGF-1. This effect may be partly explained by its contribution to endothelial integrity and maintenance of homeostasis.

The potential of IGF-1 axis-related genetic variants as a biomarker has been found to be contradictory across various cancer types, including OC [38]. This dual and context-dependent prognostic nature of the IGF-1 axis may reflect differences in the cellular origin, underlying physiology, and hormone receptor status of distinct tumour subtypes. Hence, the prognostic nature of IGF-1 expression may also vary across histological and molecular subtypes of OC, a hypothesis that warrants further investigation [18].

Given the complexity of these mechanisms, further research in larger cohorts and other populations is required. In addition, functional studies are necessary to elucidate the biological implications of *IGF1* rs6220 and *IGF1R* rs2016347, particularly how they influence the expression and/or activity of their respective proteins.

Together, our findings suggest that *IGF-1 axis*-related polymorphisms play a significant, yet context-dependent, role in ovarian tumourigenesis, with potential clinical utility as prognostic and predictive biomarkers. Furthermore, these findings highlight promising biomarkers that may contribute to the development of more effective and personalised treatment strategies for OC. Future research encompassing a larger spectrum of IGF-1axis-related genes could further refine our understanding of this pathway and support the development of targeted therapy approaches.

## Data Availability

The data presented in this study is available on request from the corresponding author.
